# 肺癌伴多发磨玻璃密度结节的多层螺旋CT表现及其临床意义

**DOI:** 10.3779/j.issn.1009-3419.2012.11.10

**Published:** 2012-11-20

**Authors:** 静 肖, 玉芬 吴, 亮 徐, 勇 黄, 聿辉 刘

**Affiliations:** 1 250031 济南，山东省医学科学院附属医院放射科 Department of Radioligy, Affiliated Hospital of Shandong Academy of Medical Sciences, Ji'nan 250031, China; 2 250117 济南，山东省肿瘤防治研究院影像科 Department of Radioligy, Tumor Hospital of Shandong Province, Ji'nan 250117, China

**Keywords:** 肺肿瘤, 磨玻璃, 非典型腺瘤样增生, 细支气管肺泡癌, 计算机断层扫描, Lung neoplasms, Atypical adenomatous hyperplasia, Bronchioloalveolar carcinoma, Focal pure ground-glass opacity, Computed tomography

## Abstract

**背景与目的:**

部分肺癌患者除原发灶外，还伴多发纯的磨玻璃影（pure ground-glass opacities, pGGO），本研究对pGGO的数目、分布、形态特征进行评估。对没有手术切除的pGGO进行CT随访，观察其变化。

**方法:**

回顾性分析25例在CT图像上伴有多发pGGO的肺癌病例。

**结果:**

pGGO的数目总共207个，最大直径2 mm-31 mm。原发灶和pGGO都可出现分叶。183（88.4%）处pGGO边缘清晰或为圆形。87处pGGO中，经外科手术切除17处病变，病理结果为：AAH 13例，BAC 3例，局灶性纤维化1例。120处随访的pGGO，CT随访的中位时间是61.5个月，113（94.2%）处病变没有变化，1处缩小，6处消失。

**结论:**

肺癌和pGGO中可以出现在相同和/或不同的叶。大多数pGGO的大小在随访期间没有变化。很多小的病变在病理上诊断为AAH和BAC。这些数据证明外科手术切除原发肿瘤对余下的pGGO的预后没有影响。

## Abstract (en)

**Background and objective:**

Some cases of lung cancer in addition to a primary tumor are associated with multiple pure ground-glass opacities (pGGOs). The objective of this study is to evaluate the clinical significance of multiple pGGOs on CT images of patients with lung cancer. The number, size, distribution, and morphological characteristics of the pGGOs were evaluated. Serial changes in pGGOs that were not surgically resected were analyzed at follow-up CT.

**Methods:**

The cases of 25 patients with proven lung cancer and associated multiple pGGOs on CT images were retrospectively reviewed.

**Results:**

In total, 207 pGGOs were detected. The size of the opacities ranged from 2 mm to 31 mm in largest diameter. Lung cancer and pGGOs were seen in the same lobe and/or in other lobes. Of the lesions, 183 (88.4%) had a round shape or well-defined border. Histological findings were obtained for 17 lesions representing 87 pGGOs that were surgically resected, namely, 13 atypical adenomatous hyperplasias, 3 bronchioloalveolar carcinomas, and 1 focal fibrosis. Of the 120 pGGOs followed up with CT for a median duration of 61.5 months, 113 (94.2%) retained their size, 1 decreased in size, and 6 disappeared.

**Conclusion:**

The size of most pGGOs associated with lung cancer did not change during the follow-up period. Most of the lesions histologically diagnosed were atypical adenomatous hyperplasias or bronchioloalveolar carcinomas. The results justify the therapeutic strategy of resecting the primary tumor without therapeutic intervention in the remaining pGGOs.

随着低剂量CT和肺癌的筛查越来越普遍，肺癌的检出率越来越高^[1]^。并且部分肺癌除原发灶外，在CT上还表现为肺内有一个或多个纯的磨玻璃影（pure ground-glass opacities, pGGO）。pGGO可能为肿瘤病变，例如非典型腺瘤样增生（atypical adenomatous hyperplasia, AAH）、细支气管肺泡癌（bronchioloalveolar carcinoma, BAC），还有极少数情况下为肺内转移。这些肿瘤性病变可影响治疗策略和临床疗效。本研究旨在探讨肺癌患者伴发多个pGGO在CT上的表现及其临床意义。

## 1 材料与方法

### 1.1 病例资料

选取山东省肿瘤医院2006年1月-2010年10月的25例患者（9例男性，16例女性，年龄43岁-81岁，平均59.8岁），均被病理证实为肺癌患者，并且在CT图像上肺内均伴发多个pGGO，不伴有任何实性成分。

### 1.2 影像学检查方法

采用Siemens Somatom Sensation 16排多层螺旋CT扫描。扫描范围自肺尖扫描至双侧膈肌。肺窗窗宽1, 600 HU，窗位-600 HU；纵隔窗窗宽400 HU，窗位40 HU。采集层厚1.5 mm，层厚5.0 mm，部分层厚2 mm-3 mm。

### 1.3 CT表现分型

根据原发灶所在肺叶把这些病变分成4型：1型，pGGO和原发灶在一个肺叶；2型，pGGO和原发灶在不同的肺叶；3型，pGGO和原发灶同时在相同和不同的肺叶；4型，pGGO和两个或多个原发肿瘤在相同和不同的肺叶。其中1型、2型、3型原发灶均只有一处。对pGGO的边界（明确界定或定义不清）和形状（圆形或多角形）的形态特征进行了评估。

## 2 结果

25例患者中，19例患者为1处原发灶，5例为2处原发灶，1例为3处原发灶。总共32处原发灶。其中23例患者的29处原发灶被切除。另外3例在CT引导下穿刺活检。32处原发性灶的组织学类型：31处为腺癌，另1处为鳞状细胞癌。根据2007年国际抗癌联盟的TNM分期，Ⅰa：18/25；Ⅰb：4/25；Ⅲa：1/25；Ⅲb：2/25。

患者的预后：初次CT检查后，2例患者于初次CT检查后24个月和34个月死于肺癌。1例患者初次CT检查的第82个月复发。其它22例均存活，并在28个月-94个月（中位数为61.5个月）随访期间无复发迹象。120处随访的pGGO，113（94.2%）处病变没有变化，1处缩小，6处消失。

CT表现：原发灶大小介于3 mm-46 mm，平均直径为21.3 mm。26处原发灶在CT上表现为混合性磨玻璃密度（mixed ground-glass opacities, mGGO），均为腺癌。6处在CT上表现为实性结节的原发灶，5例为腺癌，1例为鳞状细胞癌。

CT图像的pGGO的数量为2个-43个，平均8.5个，总数为207个。pGGO的最大径2 mm-30 mm，平均6.2 mm。207个pGGO的形态学特征，如表 1所示。183（88.4%）处的病变，有明确的边界或为圆形。手术切除其中17处病变，病理证实13处为AAH（图 1），3处为BAC，1处为纤维灶。

**1:** pGGO结节的形态特征 The morphological characteristics of the pGGO nodules

Border	Shape	*n* (%)
Well-defined	Round	140 (67.6)
	Polygonal	43 (20.8)
Ill-defined	Round	2 (1.0)
	Polygonal	22 (10.6)

**1:**
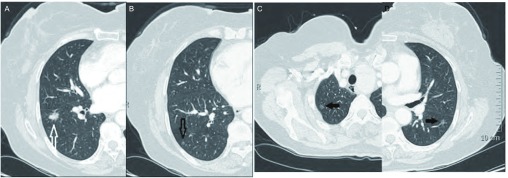
男，56岁，腺癌（3型）。A：中示右肺下叶原发灶（白色空心箭头）；B、C、D中pGGO（黑色空心箭头）与原发灶一同被切除，病理证实为AAH，另外两处pGGO（黑色实心箭头）未切除。 Male, 56 years old, adenocarcinoma (type 3). A shows the primary tumor in the right lower lobe (white hollow arrow); B, C, D show pGGO (the black hollow arrow) together with the primary tumor surgical resection, pathologically confirmed as AAH, another two pGGO (black solid arrows) is not removed.

将这些病例按照原发灶与pGGO的关系分为4种类型。根据这个CT分类，1型3例，2型4例（图 2），3型12例，4型6例。每一型病例的pGGO数量：1型，2-5（平均3.3）；2型，2-3（平均2.3）；3型，2-18（平均8.0）；4型，2-41（平均15.2）。

**2:**
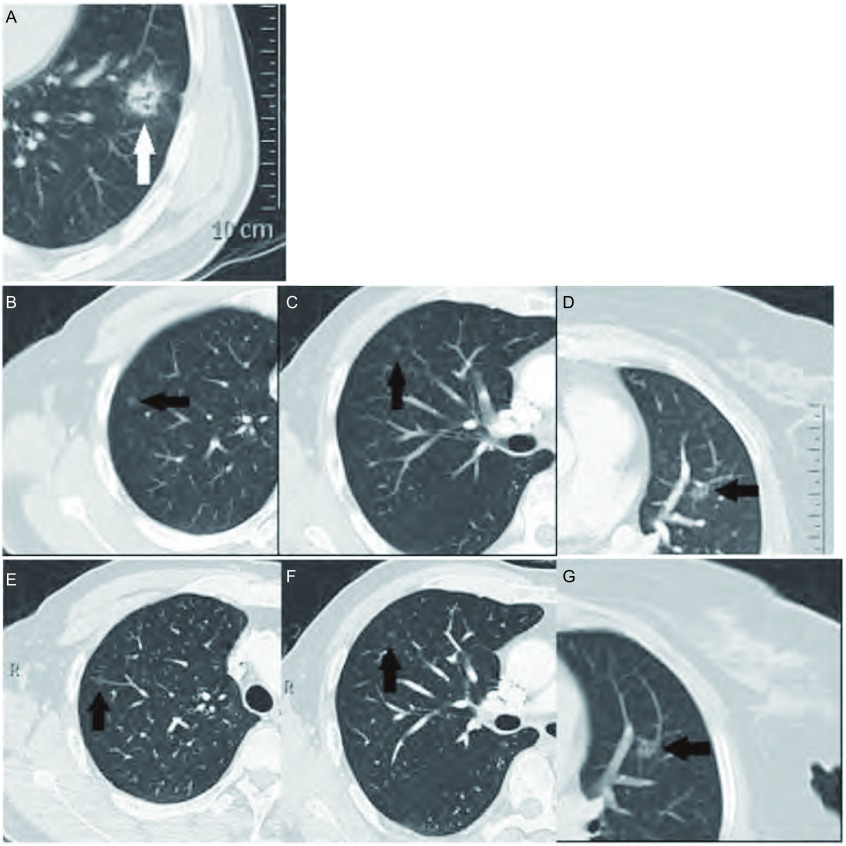
女，68岁，腺癌（2型）。A中示原发灶(白色实心箭头)位于左肺下叶；B、C、D可见到右肺上叶、中叶及左肺上叶pGGO（黑色实心箭头），E、F、G示术后CT随诊pGGO均无变化。 Female, 68 years old, adenocarcinoma (type 2). A shows primary tumor (white solid arrow) in the left lower lobe; B, C, D show right upper lobe, the mid and left upper lobe pGGO (black solidarrow), E, F, G show pGGO CT followed up after surgery no change.

## 3 讨论

CT技术的进步使表现为pGGO的小病灶检出率越来越高。pGGO是一种非特异性的发现，在各种疾病的CT上都可以表现为pGGO，包括炎症、纤维化、肿瘤性疾病。肿瘤性病变包括BAC、AAH、淋巴瘤，当其pGGO持续增大时更应该考虑肿瘤性病变^[2]^。

多发pGGO常在原发性肺癌患者的CT中偶然发现。在本研究中，88.4%的pGGO有一个明确的边界或圆形。据Nambu等^[3]^的研究，边界清晰的pGGO肿瘤性病变比非肿瘤性病变更多。所以本研究中大多数pGGO更可能是肿瘤病灶，包括BAC和AAH。

pGGO的随访变化也是评价这些病灶的一个重要因素。在我们的随访研究中，94.2%的pGGO的大小没有改变。有研究^[4]^显示，与肺癌患者相关的pGGO随访期间并没有改变。在本研究中，1处pGGO病灶缩小，6处pGGO病灶消失。所有这7处pGGO在HRCT图像上表现为多边形形状，这表明它们是炎性病变。肺结节已被普遍认为如果在两年观察期内大小不变或者缩小表明为良性结节^[5]^。更长的随访时间是必需的，但理想随访期间确认良性病变的性质仍有争议。据报道^[6]^，如果pGGO结节增大，或在随访期间其内出现实性成分，应及时进行处理，包括切除（侧或楔形切除术）和立体定向照射^[7]^。

本研究主要基于pGGO与原发肿瘤的位置和分布的局部性病灶分为4个类型。根据这一分类，大约一半的病例3型，即pGGO与原发肿瘤在相同与不同的叶。在本研究中，与肺癌在不同肺叶的只有一个AAH病理被证实。一些报道^[8]^所描述的与肺癌相关的多个AAH，这些AAH大部分发现于原发肿瘤切除病理标本。应当指出，肺癌和pGGO可以在同一肺叶中被发现。

虽然有学者研究^[9]^多个原发性肺癌成功切除，基于手术结果，但没有进行适当的治疗。我们在CT分类体系的基础上，可以提出适当的治疗策略。1型建议单肺叶切除，因为原发肿瘤和pGGO在同一肺叶。2型或3型也可进行单肺叶切除，未切除的pGGO可以稳定很长时间，故可行观察。有报道^[4]^称，大多数与肺癌相关的pGGO在随访期间大小没有变化。Kim等^[10]^报道，与原发灶共存的AAH或BAC患者预后可能完全取决于原发灶，而不是AAH或BAC。另一种选择是可以在另一个肺叶对pGGO行附加段或楔形切除术。4型的治疗策略取决于多个原发肿瘤的分布。如果所有原发肿瘤在相同的叶，pGGO在其它叶视为类型2型或3型，可以行单一肺叶切除。当原发肿瘤是在多个叶，结合肺叶、段切除和楔形切除术是必要的^[9]^。另一种选择是对这些肿瘤进行立体定向放射。即使在这种情况下，pGGO也可密切随访，不用治疗。

综上所述，肺癌和pGGO中可以出现在相同和/或不同的叶。大多数pGGO在随访期间大小没有变化。为数不多的病变组织学诊断，大部分为AAH和BAC。本研究表明切除原发性肿瘤的治疗策略对余下的pGGO预后没有影响。
